# Spatial and Temporal Patterns of Ross River Virus in Queensland, 2001–2020

**DOI:** 10.3390/tropicalmed6030145

**Published:** 2021-08-03

**Authors:** Wei Qian, Cameron Hurst, Kathryn Glass, David Harley, Elvina Viennet

**Affiliations:** 1 UQ Centre for Clinical Research, The University of Queensland, Herston, QLD 4059, Australia; wei.qian@uq.net.au (W.Q.); d.harley@uq.edu.au (D.H.); 2 Department of Statistics, QIMR Berghofer Medical Research Institute, Brisbane, QLD 4006, Australia; cameron.hurst@qimrberghofer.edu.au; 3 Research School of Population Health, Australian National University, Acton, ACT 2601, Australia; Kathryn.Glass@anu.edu.au; 4 Institute for Health and Biomedical Innovation, School of Biomedical Sciences, Queensland University of Technology, Kelvin Grove, QLD 4059, Australia; 5 Clinical Services and Research, The Australian Red Cross Lifeblood, Kelvin Grove, QLD 4059, Australia

**Keywords:** epidemiology, Ross River virus, spatial pattern, temporal pattern, disease burden

## Abstract

Ross River virus (RRV), the most common human arbovirus infection in Australia, causes significant morbidity and substantial medical costs. About half of Australian cases occur in Queensland. We describe the spatial and temporal patterns of RRV disease in Queensland over the past two decades. RRV notifications, human population data, and weather data from 2001 to 2020 were analysed by the Statistical Area Level 2 (SA2) area. Spatial interpolation or linear extrapolation were used for missing weather values and the estimated population in 2020, respectively. Notifications and incidence rates were analysed through space and time. During the study period, there were 43,699 notifications in Queensland. The highest annual number of notifications was recorded in 2015 (6182), followed by 2020 (3160). The average annual incidence rate was 5 per 10,000 people and the peak period for RRV notifications was March to May. Generally, SA2 areas in northern Queensland had higher numbers of notifications and higher incidence rates than SA2 areas in southern Queensland. The SA2 areas with high incidence rates were in east coastal areas and western Queensland. The timely prediction may aid disease prevention and routine vector control programs, and RRV management plans are important for these areas.

## 1. Introduction

Ross River virus (RRV) is the most widespread mosquito-borne disease in Australia and causes arthritis, rash, and constitutional symptoms (fever, fatigue, and myalgia) [1]. An average of 4653 cases have been recorded in Australia annually since 1993 [2], leading to a significant public health and economic burden. Nearly half (49.0%, 63,880/130,271) of these Australian cases occurred in Queensland [2].

Ross River virus is an *Alphavirus* from the *Togaviridae* family and is transmitted to humans by mosquitoes. Important Queensland vector species include *Culex annulirostris*, which breeds in freshwater habitats, *Aedes vigilax*, which breeds in mangroves and salt marshes, and *Aedes notoscriptus*, which breeds in artificial containers. Other vector species include *Aedes camptorhynchus*, *Culex australicus*, *Anopheles annulipes*, and *Culex globocoxitus*, although these are not considered to be the main species that contribute to RRV transmission [3]. Non-human reservoir hosts include marsupials, placental mammals, and birds [4]. Ross River virus transmission is influenced by mosquito abundance and host populations, and climatic (e.g., rainfall, temperature), environmental (e.g., river flow, and vegetation cover) and socio-economic factors (e.g., urban development and housing infrastructure) [5]. The humid and warm climate in most areas of Queensland is conducive to mosquito breeding, adult survival, and consequently disease transmission.

Ross River virus has been nationally notifiable in Australia since 1991 [6]. Laboratory evidence is required to notify a RRV case. A revised case definition for RRV was implemented following 1 January 2013 in response to known cross-reaction between RRV IgM and the IgM response to other pathogens [7]. A further revised definition was applied following 1 January 2016 to remove a single IgM positive test result as a diagnostic criterion. Currently, a confirmed case requires virus isolation, virus detection by nucleic acid testing, or IgG seroconversion or a significant increase in IgG antibody level [8]. A probable case is defined as detection of RRV IgM and IgG, except when IgG has also been detected more than 3 months previously. Both confirmed cases and probable cases are notified [8]. These notification data are the basis for published descriptive and analytic studies of RRV.

Some studies have explored the epidemiological patterns of RRV in Queensland. Gatton et al. [9] first described RRV incidence across Queensland from 1991 to 2001 and applied cluster analysis to identify outbreak patterns. The temporal trends, spatial variation, and spatial–time clustering of RRV in Queensland from 2001 to 2011 were analysed by Yu et al. [10]. Murphy et al. [11] presented temporal and spatial patterns and hot spots of RRV in southeast Queensland from 2001 to 2016. Exploring temporal and spatial patterns of RRV in Queensland over a long period may identify high-risk areas and possible risk factors for these areas. Weather is associated with RRV incidence and outbreaks as demonstrated by some studies conducted across Australia [12,13,14,15]. However, few studies have analysed weather patterns in high-risk areas over a long period.

Here we present a retrospective descriptive analysis of RRV in Queensland, Australia. We aim to (1) describe the temporal trends and spatial patterns of RRV in Queensland over the past two decades; (2) identify time periods with large numbers of notifications and areas with high incidence rates for targeted surveillance and control; and (3) summarise weather patterns in areas with high case numbers or high incidence. The results will support RRV disease prevention and health promotion, and guide health resource allocation in Queensland.

## 2. Materials and Methods

### 2.1. Study Area

This study was conducted in Queensland, Australia, which has an area of 1,727,000 square kilometres and a population of 3,571,469 in 2001 and 5,174,400 in 2020 [16,17]. Queensland is divided into 528 Statistical Area Level 2 (SA2) units and 82 Statistical Area Level 3 (SA3) units [18] which vary in their population density and natural environment. Northern Queensland and coastal areas from Townsville to Mackay have a humid subtropical climate with high humidity in the summer and a warm winter. Most inland areas are characterised by a hot semi-arid climate with a hot dry summer and warm winter, while coastal areas south of Mackay have a warm humid summer and mild winter [19,20]. 

### 2.2. Data Collection and Collation

Data for notified RRV cases in Queensland between 1 January 2001 and 31 December 2020 were acquired from the Queensland Department of Health. These data were in the form of daily de-identified records at the SA2 level with the date of report. Annual human population estimates for each SA2 area during the study period were obtained from the Australian Bureau of Statistics [21]. Daily rainfall, maximum temperature, and minimum temperature data were obtained from the Australian Bureau of Meteorology [22]. 

Weekly and yearly total RRV notifications were calculated for each SA2 and SA3 area. The SA2 area population for 2020 was estimated using linear extrapolation from the population in previous years. The annual incidence rates were calculated as the number of notifications divided by annual average number of residents at the SA2 and SA3 level. Seasonal incidence rates were calculated as the total number of notifications by season divided by the average population across the study period in each SA2 area. Cumulative incidence was calculated as the total cases over the study period divided by the average population across the study period in each SA2 and SA3 area or in Queensland. Averages for daily weather data by SA2 level were calculated by averaging all data points in the grid maps within each SA2 area. Spatial interpolation using adjacent points was applied to areas that did not contain any weather data points. Average daily temperature was calculated as the mean of maximum and minimum temperatures. The number of rainy days and number of days with rainfall over 15 mm were calculated for each SA2. Notified RRV cases, incidence rates, human population, and weather data were linked at each SA2 or SA3 area.

### 2.3. Descriptive Analysis

Temporal trends were used to describe the weekly distribution of RRV notifications and peak years in Queensland during the study period. Incidence rates were calculated to adjust for changing population sizes over time or differences by area. Spatial trends in the total cases and cumulative incidence were mapped to show the distribution of RRV over the past 20 years. Spatial units for descriptive analyses were SA2 areas. When presenting RRV notifications and incidence rates in heat maps, SA3 areas were applied because it is difficult to present all 528 SA2 areas clearly in one figure.

Hot spot analysis of RRV incidence during the study period was conducted based on a fixed distance band. Spatial autocorrelation using Global Moran’s I and cluster and outlier analysis using Local Moran’s I were conducted. Both methods aim to detect spatial patterns of distribution of RRV incidence rates. The former indicates clusters that have similar incidence rates in adjacent areas, either high or low incidence. The latter highlights areas with similarly high or low spatial autocorrelation, evaluated by Local Moran’s I, with surrounding areas. Figures were produced to show hot spots and clusters of RRV incidence in Queensland.

The coastal areas in this study were defined as SA2 areas with at least one South Pacific Ocean boundary. The coastal and non-coastal SA2 areas in Queensland were categorised into four groups: (1) those with the highest 10% of RRV notifications; (2) those with the highest 10% of RRV incidence rates; (3) those with the lowest 10% of RRV notifications or incidence rates; and (4) all the other areas. Areas with low numbers of notifications typically also had low incidence rates and thus we combined these areas into a single group. The average daily temperature, daily rainfall, and the number of rainy days in spring (September to November), summer (December to February the following year), autumn (March to May), and winter (June to August) were listed to show the weather patterns of areas in these four groups. 

All analyses and figures were performed and produced with R 3.6.3 (R Core Team, Vienna, Austria) [23] and ArcMap 10.7.1 (Environmental Systems Research Institute, Redlands, CA, USA) [24] using GCS_GDA_1994 Geographic Coordinate Systems. This study was approved by the University of Queensland Human Research Ethics Committee A (2019/HE002772).

## 3. Results

A total of 43,699 cases were reported in Queensland, Australia, from 2001 to 2020. The highest number of notifications and highest incidence rate were recorded in 2015 (6161 notifications, 12.9 notified cases per 10,000 people), while the lowest number of notifications (883) and incidence rate (2.4 per 10,000 people) were reported in 2002 (Table S1). Over the study period, the mean annual incidence rate was 5 per 10,000 people. Around 84 per 10,000 people (43,699 total cases/5,174,400 population in 2020) were infected with RRV over the 20-year study period.

### 3.1. Temporal Trends

**Figure 1:**
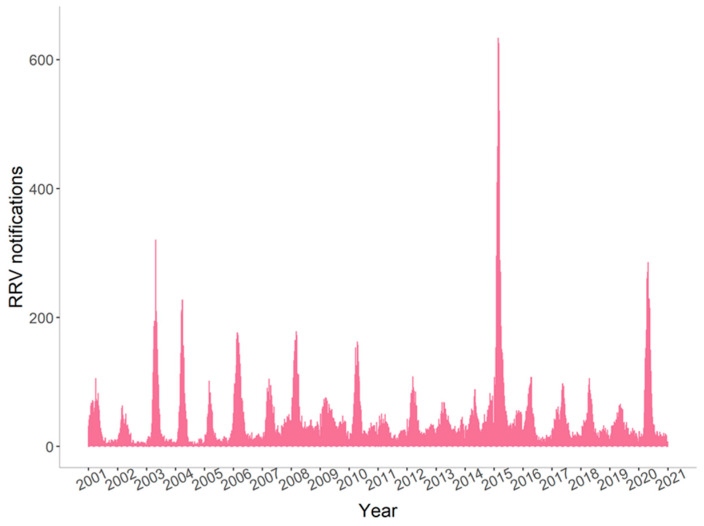
Weekly Ross River virus notifications in Queensland, 2001–2020.

RRV cases reported in winter and spring accounted for 26.1% (11,411/43,699) of all cases. Fewer RRV cases were reported in winter and spring before 2006 (Figure 1 and Table S1). Since 2006, RRV cases have been reported throughout the year. There were three notable peaks in notifications during the study period in which weekly cases rose above 250: 28 April to 4 May in 2003; 2 February to 29 March in 2015; and 13 April to 3 May in 2020.

**Figure 2:**
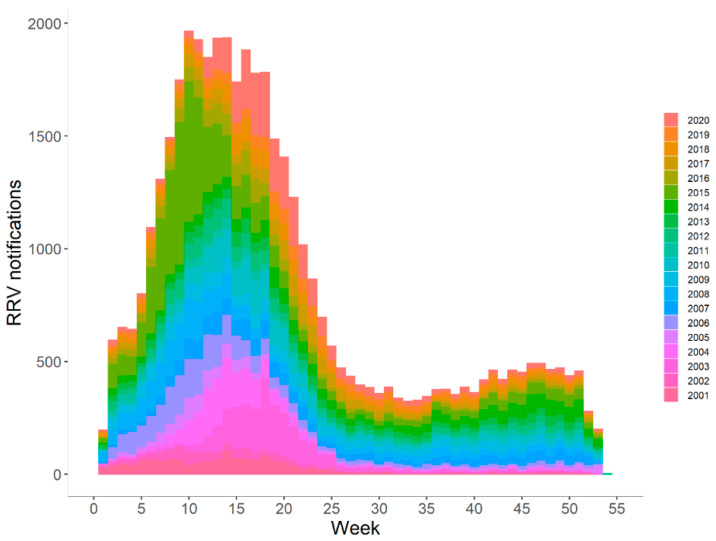
Weekly cumulative Ross River virus notifications in Queensland in each year, 2001–2020.

RRV notifications were highly seasonal with 63.4% of all cases (27,689/43,699), notified from February to May (Figure 2). The peaks mainly occurred in several weeks from March to May. In 2015, notifications from January to May accounted for 80.5% (4977/6182) of all notifications across the year. A minor rise in total cases observed in spring indicated an ongoing risk of RRV infection throughout the year.

### 3.2. Spatial Patterns

**Figure 3:**
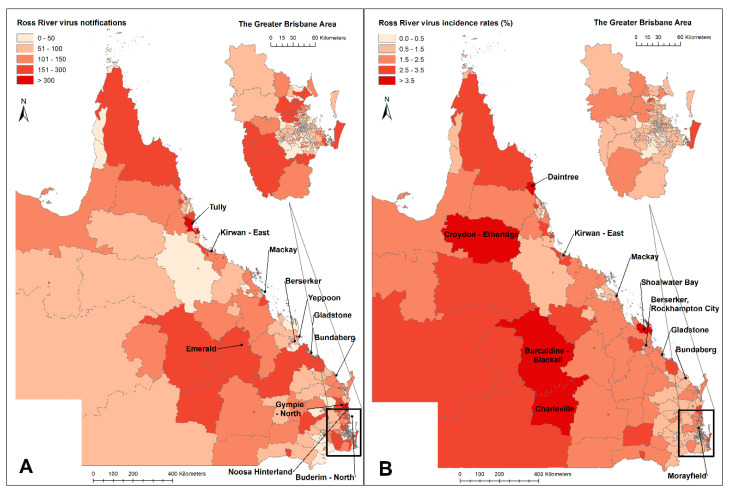
Spatial distribution of Ross River virus total notifications (**A**) and cumulative incidence (**B**) in Queensland, 2001–2020. SA2 areas with over 300 notifications and cumulative incidence over 350 cases per a population 10,000 are named in the figure.

Figure 3 shows that high total numbers of notified RRV cases were reported in some coastal areas (e.g., Tully; for others, see Figure 3) and in some inland areas (e.g., Emerald). Some SA2 areas in coastal areas (e.g., Daintree) and western Queensland (e.g., Charleville) reported high incidence rates (>350 cases per a population of 10,000 over the study period). Southeast Queensland, especially Brisbane, had lower incidence in general than other areas. The average cumulative incidence of the Greater Brisbane area over the whole study was around 70 cases per 10,000 people. Yearly spatial distributions of RRV notifications and incidence rates in Queensland are provided in Figures S1 and S2. Some inland areas tend to have relatively high incidence rates throughout the year, while the coastal areas mainly have high incidence rates in summer and autumn (Figure S3). The Global Moran’s I is 0.045 with statistical significance which shows a weak spatial autocorrelation. Hot spots and clusters of RRV incidence are mainly located in central and north Queensland, and cold spots are in south-east Queensland (Figures S4 and S5).

**Figure 4:**
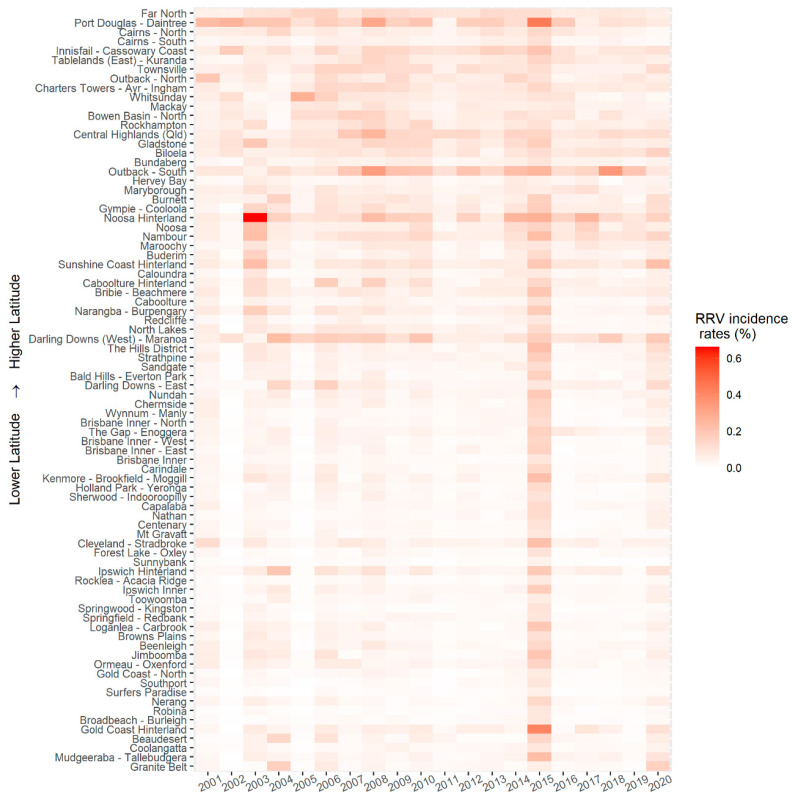
Yearly Ross River virus incidence rates in SA3 areas of Queensland from 2001–2020 (ordered by latitude).

The heat map in Figure 4 displays the yearly incidence rates of RRV in each SA3 area, ordered by latitude of the centroid. Generally, northern Queensland has higher incidence rates and higher numbers of notifications (Figure S6) than southern Queensland. In some SA3 areas such as Daintree, Noosa Hinterland, Outback-South, and Darling Downs, the incidence rates tend to be higher than in other areas throughout the whole study period. In Noosa Hinterland, there was a high incidence rate in 2003. In 2015, higher incidence rates were observed across Queensland areas than in the other years. High incidence of RRV was reported inland and along the coast of Queensland (Figure S7).

### 3.3. Weather Conditions

To further investigate weather conditions within the SA2 areas with high numbers of cases or high incidence rates, we provided the mean daily rainfall and daily temperature in these areas during the study period (Table 1 and Figure S8). Higher spring and summer temperatures were observed in areas in which notifications’ numbers and incidence rates were higher than in other areas. Coastal areas with high incidence rates or high notifications had more rainfall and rainy days in summer than other areas during the study period. Non-coastal areas with high incidence rates had much lower daily rainfalls and fewer days with heavy rains (daily rainfall > 15 mm) but more rainy days than average. Table 1 also illustrates that coastal and non-coastal areas with high incidence rates have different weather patterns. Compared to non-coastal areas with high incidence rates, coastal areas with high incidence rates were cooler in summer and warmer in winter, experiencing more rainfall throughout the year and more days of heavy rain in summer and autumn.

## 4. Discussion

In this study, we described the temporal and spatial patterns of RRV at the SA2 level in Queensland over the past two decades. We found that RRV is actively transmitted in many areas of Queensland. The high numbers of notifications recorded in peak years and high proportion of RRV cases in the off-season indicate the possibility of outbreaks and disease transmission outside the usual peak periods. There is an annual peak in autumn. Many coastal areas and some inland areas have high numbers of notifications or high incidence rates. This descriptive analysis demonstrates shifts in the seasonality of transmission including high transmission rates early in the year and increased transmission throughout the year.

The case definition for RRV was revised in 2013 and 2016. The prior use of single IgM positive tests for RRV led to over-diagnosis and possible false-positive diagnostic test results, especially during the off-season [25,26]. The removal of these results may lessen the overestimation of disease burden; however, it means that comparisons of RRV notifications before and after 2016 should be interpreted cautiously. 

RRV notifications in the 2015 outbreak were widely spread across Queensland with the peak period from mid-February to mid-March. Notifications from January to May accounted for 80.5% (4977/6182) of all notifications across the year. Jansen et al. [27] suggested that a combination of ecologic factors including above-average rainfall, possible ubiquitous non-human hosts, and a widespread high abundance of mosquitoes (especially *C. annulirostris* and *Ae. procax*) may have contributed to this widespread outbreak. 

RRV notifications in 2020 were higher than in previous years and the peak period lasted longer than in other years. Global heating and heat islands in cities may be extending the period of the year when mosquito numbers are high [28]. People may also be more aware of symptoms of fever and fatigue during the world-wide pandemic of COVID-19. Population behaviour changed during March to May in that year, the usual peak season of RRV infection. People may have avoided urban areas where there is less vegetation cover, stayed at home more often, and may have spent more time gardening and exercising outdoors, all of which likely altered RRV transmission risk. However, a single year of data is insufficient to assess the effect of COVID-19 on RRV transmission patterns.

Some northern coastal areas of Queensland (Townsville, Mackay, Gladstone, Berserker, Bundaberg, and Sunshine Coast) had both high total notifications and high cumulative incidence throughout the study period. All these places are in humid and warm tropical or subtropical areas with a high population density. Some disease control strategies have been implemented in these areas: many local governments carried out mosquito control programs with average expenses from $9500 to $300,000 AUD during 1992–2004 [29]. Both saltwater and freshwater mosquito control were associated with lower RRV notifications in most of these areas [30]. 

Although south-east Queensland, including Brisbane and the Gold Coast, had a comparatively high population density, the notifications and incidence rates were generally low. This might be associated with mosquito control programs, more intensive use of such programs, and less vegetation cover in these areas than elsewhere in Queensland [31]. The Brisbane City Council and Gold Coast City Council spent around $3,000,000 and $1,000,000 AUD each year, respectively, on conducting freshwater and saltwater mosquito control and routine surveillance year-round from 1993 to 2004 [29]. However, south-east Queensland still experienced two periods of heightened notification numbers in 2015 and 2020. Timely prediction of possible outbreaks in these areas would be valuable.

As a passive surveillance system, RRV surveillance may be biased. Underestimation arises because some infections are asymptomatic [4,32], not all cases seek medical help, and not all patients are tested. In addition, there were false-positive diagnostic test results [26] particularly during the period that a single IgM positive test was considered diagnostic for notification purposes. A single positive IgM is not expected to correspond exclusively to recent RRV infection. Persistence of IgM for up to a year implies that old infections can be misclassified, while cross-reactivity implies that other infections may be misclassified as RRV. Although these are acknowledged issues in population-level RRV research, it is beyond the scope of our research to quantify misclassification. During outbreak years, people may be more aware of the disease symptoms and more cases might be reported [33]. Symptoms usually have their onset 3–11 days after infection [34]. 

We analysed RRV cases in each SA2 area but were unable to analyse imported cases or population mobility across areas. Notifications record the place of residence but not necessarily the location of disease acquisition. Some notified cases may have been acquired outside the reported SA2 area and this could cause large variation in estimating incidence rates in low population density areas such as in western Queensland. The RRV data is aggregated by SA2 areas, thus, the hot spot analysis and cluster analyses, which are usually applied to point data with scattered locations, have a limited scope for interpretation in this context.

RRV was more prevalent in northern Queensland and in some coastal areas than in other areas. The humid and warm weather, high vegetation density, and abundance of reservoir hosts in those areas are suitable for mosquito breeding and virus transmission [35,36,37]. However, some areas in western Queensland with hot and dry weather also had high incidence. Numerous rainy days in spring and summer, which increase mosquito breeding, might explain RRV transmission in these high incidence inland areas. The varying weather patterns in high incidence areas suggests that weather may influence RRV transmission in different ways through interaction with other disease associations (e.g., human demography, distribution and abundance of reservoir hosts, and local variation in vector ecology). An analysis at the regional scale on the associations of weather exposures and RRV transmission is necessary.

## 5. Conclusions

RRV outbreaks occur regularly in Queensland. South-east Queensland (including Brisbane and Gold Coast areas) had low incidence rates in general over 2001–2020. Incidence rates in north-eastern coastal areas and some western inland areas were higher than in south-eastern areas. Timely prediction will aid disease prevention. To improve disease control, routine vector control programs and RRV management plans are important in high incidence areas. 

## Figures and Tables

**Table 1:** The seasonal temperature and rainfall in SA2 areas with high numbers of cases or high incidence rates.

	SA2 * Areas with High Numbers of Cases		SA2 Areas with High Incidence Rates		SA2 Areas with Low Numbers of Cases or Low Incidence Rates		Other SA2 Areas	
Coastal Areas	Non-coastal	Coastal	Non-coastal	Coastal	Non-coastal	Coastal	Non-coastal	Coastal
Number of SA2 areas	17	36	15	38	29	37	223	160
Average total cases per SA2	242.5	267.1	191.0	202.3	12.2	8.9	66.1	82.7
Average cumulative incidence per SA2 (per a population of 10,000)	223.2	291.8	284.5	391.8	13.9	85.9	79.2	122.3
Average daily temperature maxima and minima through seasons **, ℃,								
Spring	(28.0, 14.3)	(28.3, 17.6)	(30.0, 15.3)	(29.5, 18.4)	(26.5, 14.0)	(27.6, 17.3)	(27.1, 14.7)	(27.8, 17.3)
Summer	(31.2, 19.9)	(30.8, 22.1)	(33.7, 21.3)	(31.6, 22.7)	(29.6, 19.4)	(30.3, 21.8)	(30.2, 20.1)	(30.4, 21.9)
Autumn	(26.7, 14.8)	(27.7, 18.4)	(28.3, 15.2)	(28.5, 19.0)	(25.7, 14.7)	(27.2, 18.1)	(26.3, 15.4)	(27.3, 18.2)
Winter	(21.5, 8.0)	(23.6, 12.5)	(22.2, 7.8)	(24.6, 13.1)	(20.9, 8.1)	(23.1, 12.3)	(21.9, 8.9)	(23.2, 12.3)
Average daily rainfall, mm, mean (SD)								
Spring	1.9 (6.1)	1.9 (6.9)	1.4 (4.6)	1.6 (6.0)	2.2 (7.2)	2.0 (7.7)	2.1 (7.0)	2.1 (7.7)
Summer	4.3 (11.8)	6.9 (18.2)	3.3 (8.7)	7.1 (17.9)	4.5 (13.4)	6.7 (18.4)	4.5 (13.1)	6.9 (18.3)
Autumn	2.5 (8.7)	4.2 (12.9)	1.9 (6.7)	3.9 (12.2)	2.6 (9.7)	3.9 (12.4)	2.6 (10.1)	4.5 (13.7)
Winter	1.3 (5.4)	1.6 (6.4)	1.0 (4.2)	1.3 (5.5)	1.4 (5.8)	1.6 (7.0)	1.4 (5.9)	1.7 (6.9)
Average days having rainfall > 0 mm/>15 mm in a year								
Spring	777/68	677/67	782/46	692/52	594/79	591/68	643/76	644/69
Summer	1052/147	1085/224	1110/108	1184/230	826/155	977/217	886/155	1028/224
Autumn	951/78	1007/130	851/59	1009/122	751/77	880/123	811/75	956/134
Winter	724/40	676/48	683/31	633/40	507/44	553/51	554/43	624/53

* SA2 = Statistical Area Level 2. The coastal and non-coastal SA2 areas in Queensland were categorized into four groups: (1) those with the highest 10% of RRV notifications; (2) those with the highest 10% of RRV incidence rates; (3) those with the lowest 10% of RRV notifications or incidence rates; and (4) all the other areas. ** Spring is from September to November; summer is from December to February in the following year; autumn is from March to May; and winter is from June to August.

## Data Availability

The data presented in this study are available in supplementary materials.

## Supplementary Materials

The following are available online at https://www.mdpi.com/article/10.3390/tropicalmed6030145/s1: Table S1: Yearly Ross River virus notifications and incidence rates in Queensland, 2001–2020; Figure S1: Yearly spatial distribution of Ross River virus notifications in Queensland, 2001–2020; Figure S2: Yearly spatial distribution of Ross River virus incidence rates in Queensland, 2001–2020; Figure S3. Spatial distribution of Ross River virus incidence by season in Queensland, 2001–2020, seasonal incidence rates were calculated as the total number of notifications by season divided by the average population across the study period in each SA2 area; Figure S4. Hot spots and cold spots of RRV incidence in Queensland, hot spots and cold spots are statistically significant spatial clusters with high values and low values of the Getis–Ord Gi* statistic, respectively; Figure S5. Clusters and outliers of RRV incidence in Queensland, clusters and outliers are statistically significant spatial clusters with similar values and dissimilar values of the Local Moran’s I statistic, respectively; Figure S6: Yearly Ross River virus notifications in SA3 areas of Queensland, 2001–2020 (ordered by latitude); Figure S7. Yearly Ross River virus incidence rates in SA3 areas of Queensland, 2001–2020 (ordered by distance to coast); and Figure S8: Average daily rainfall (mm, A) and daily temperature (℃, B) in SA2 areas of Queensland, 2001–2020.
