# Supplementary material for: Spatial and Temporal Patterns of Ross River Virus in Queensland, 2001–2020
Source: Trop Med Infect Dis. 2021 Aug 3;6(3):145. doi: 10.3390/tropicalmed6030145 (PMC8396220; doi:10.3390/tropicalmed6030145)
Supplement: Supplementary file 1 [file tropicalmed-06-00145-s001.zip › tropicalmed-1280251-supplementary.pdf]

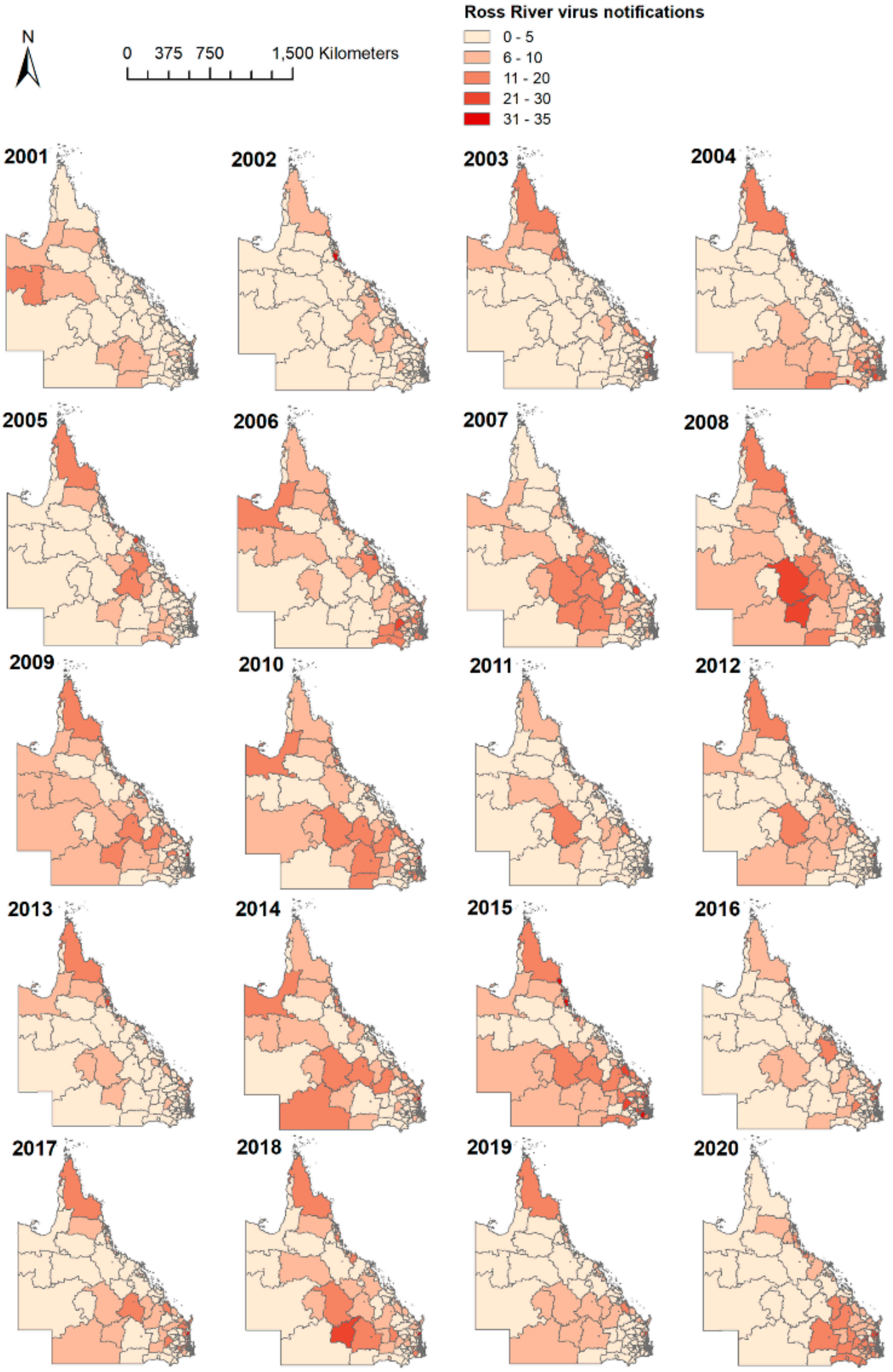

Figure S1. Yearly spatial distribution of Ross River virus notifications in Queensland, 2001–2020;.

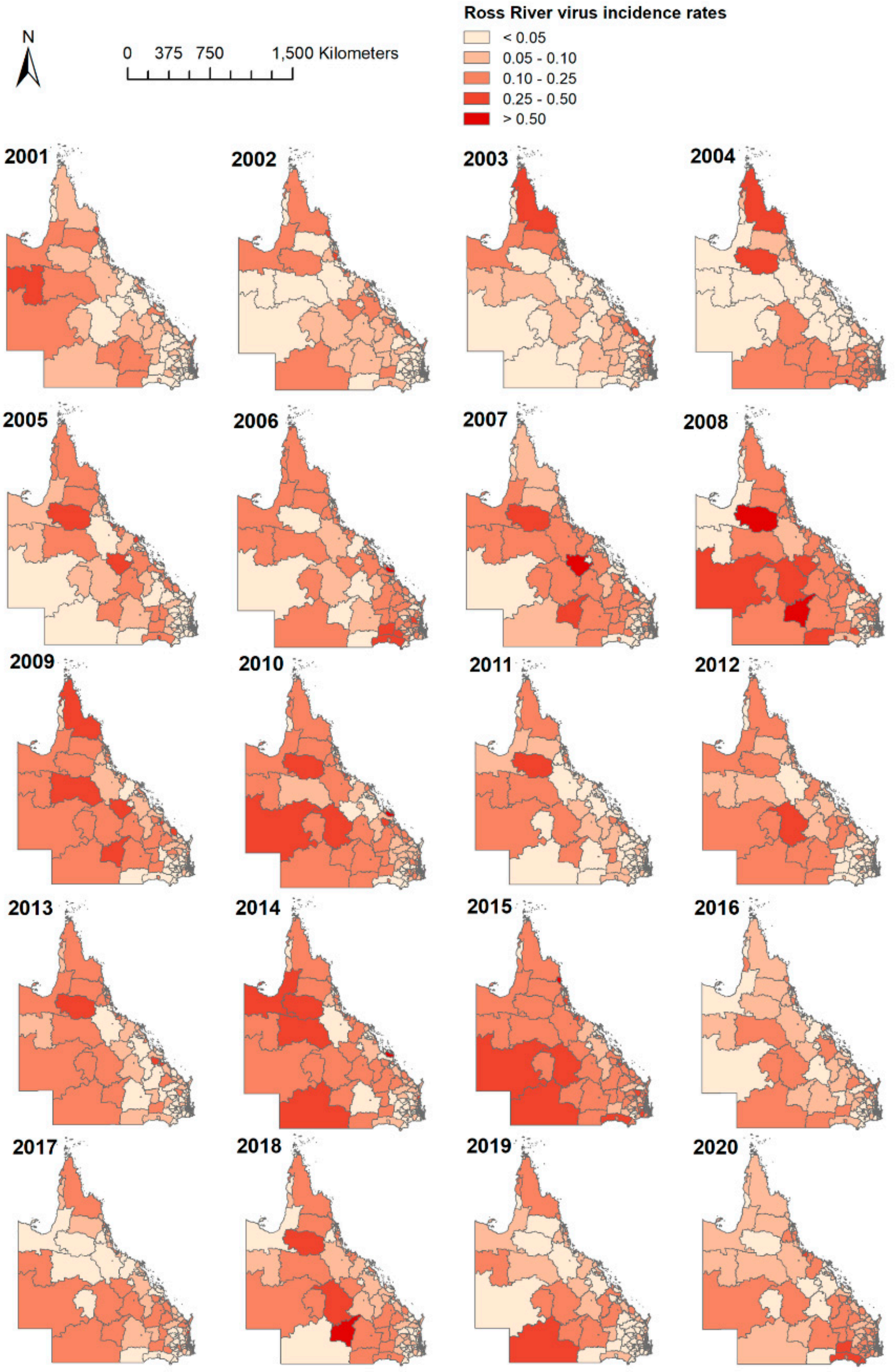

Figure S2. Yearly spatial distribution of Ross River virus incidence rates in Queensland, 2001–2020;

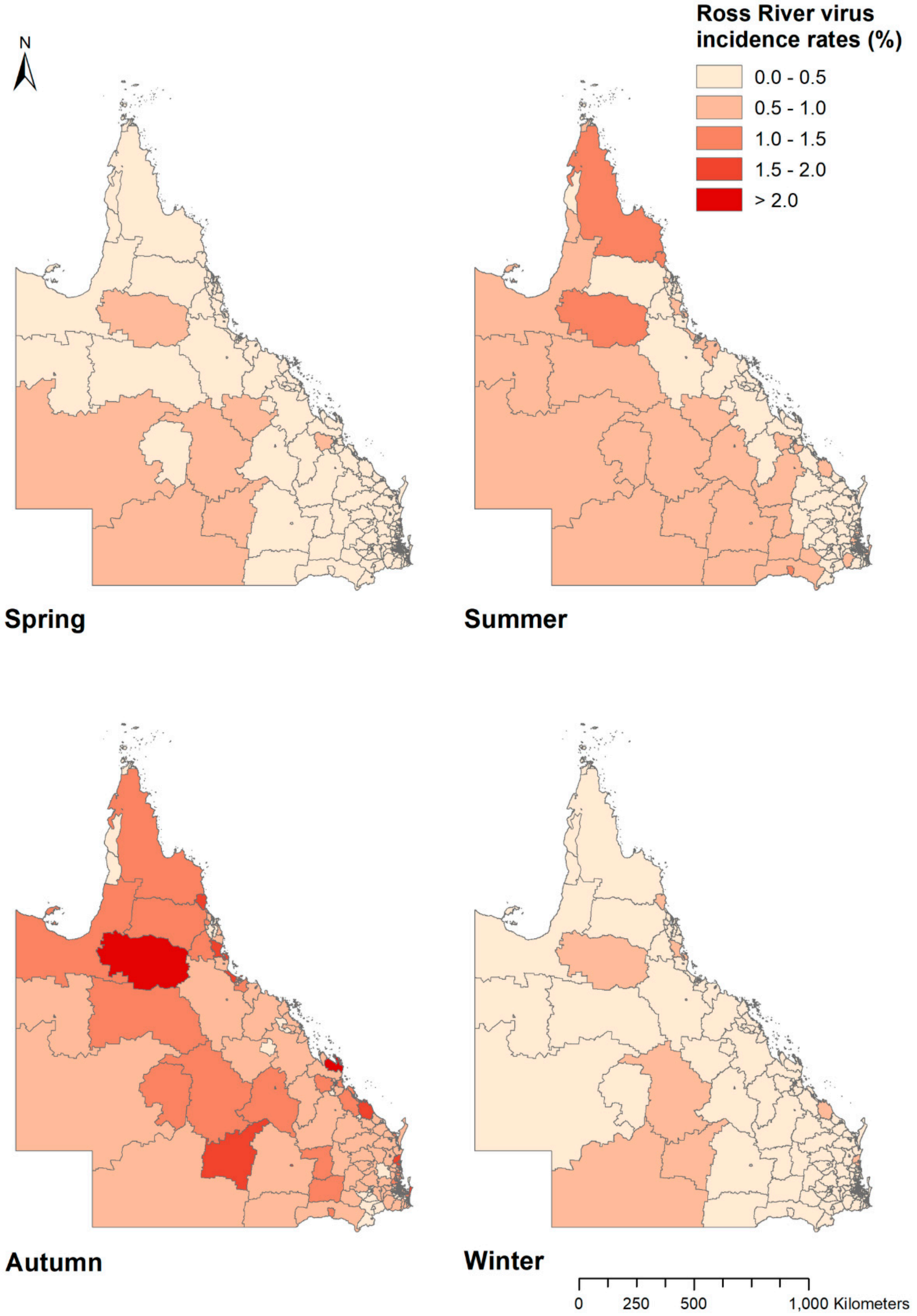

Figure S3. Spatial distribution of Ross River virus incidence by season in Queensland, 2001–2020, seasonal incidence rates were calculated as the total number of notifications by season divided by the average population across the study period in each SA2 area

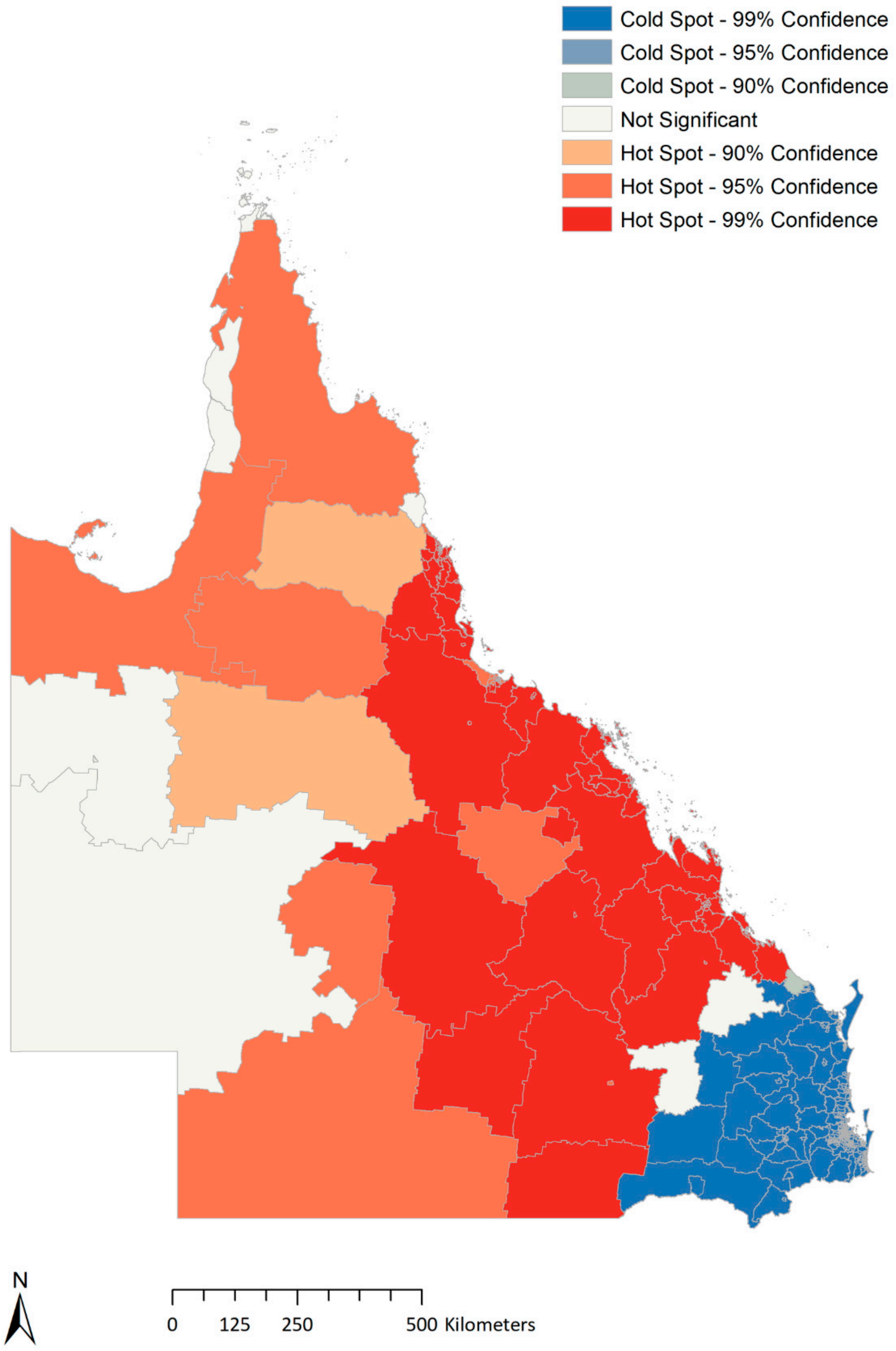

Figure S4. Hot spots and cold spots of RRV incidence in Queensland, hot spots and cold spots are statistically significant spatial clusters with high values and low values of the Getis–Ord Gi* statistic, respectively

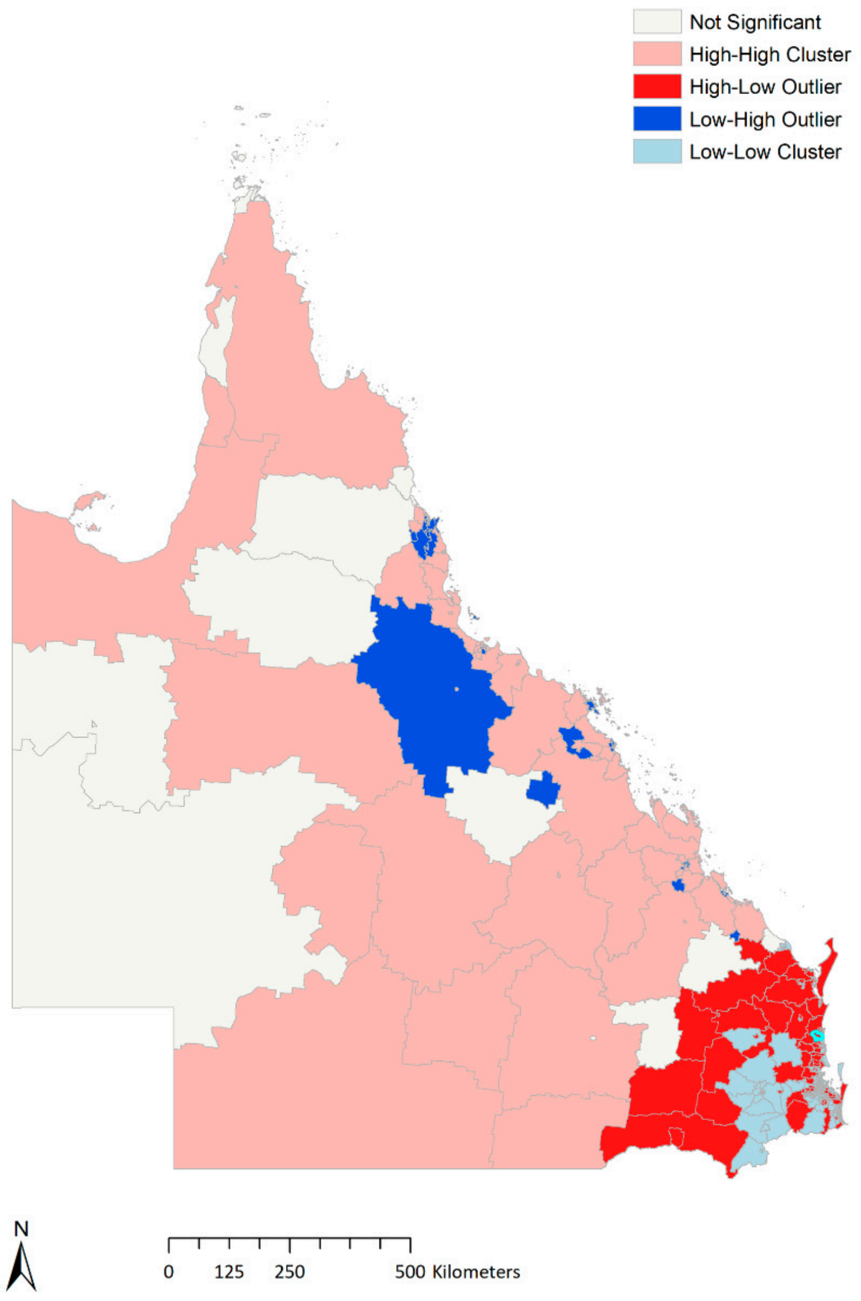

Figure S5. Clusters and outliers of RRV incidence in Queensland, clusters and outliers are statistically significant spatial clusters with similar values and dissimilar values of the Local Moran's I statistic, respectively

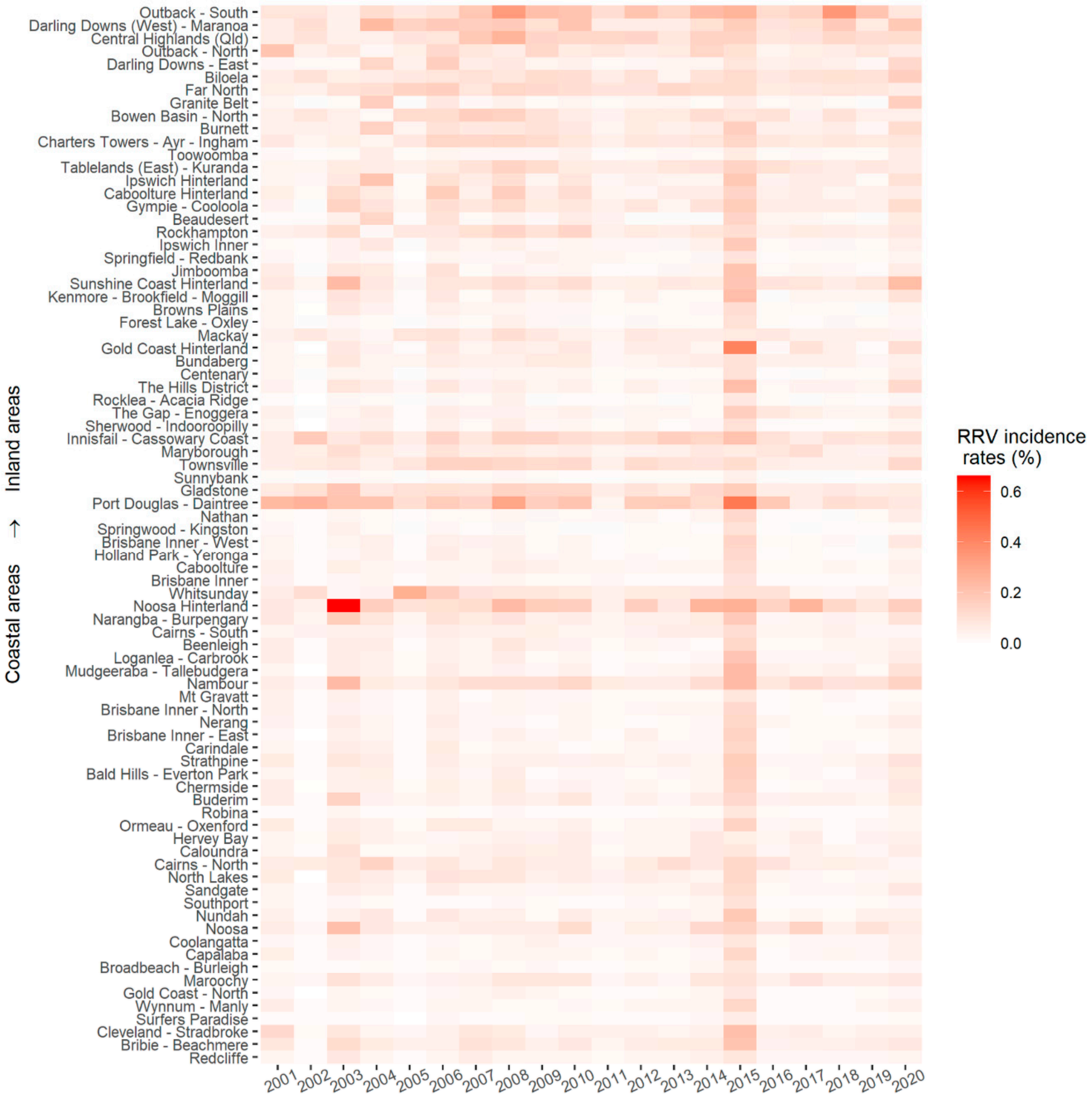

Figure S6: Yearly Ross River virus notifications in SA3 areas of Queensland, 2001–2020 (ordered by latitude);

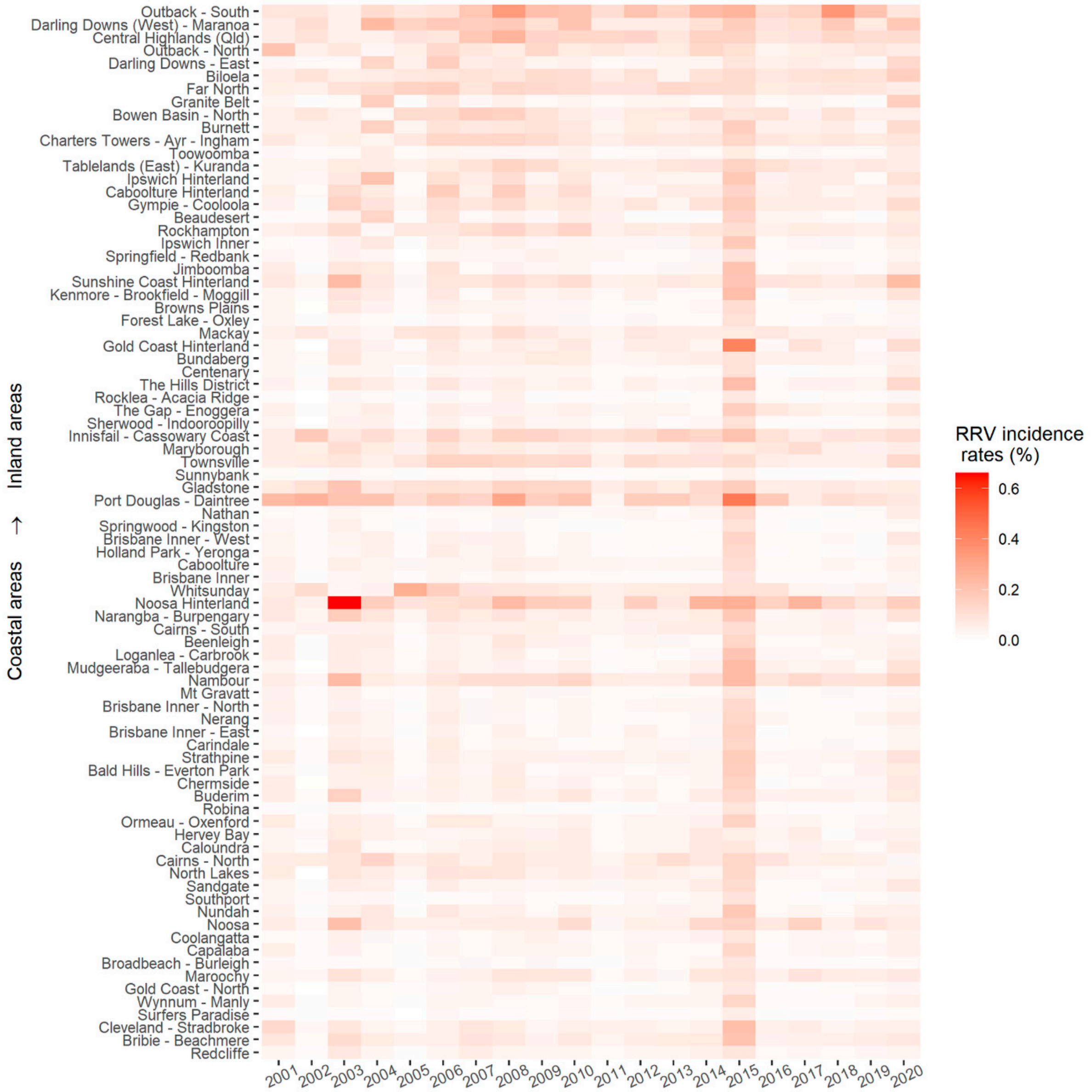

Figure S7. Yearly Ross River virus incidence rates in SA3 areas of Queensland, 2001–2020 (ordered by distance to coast); and

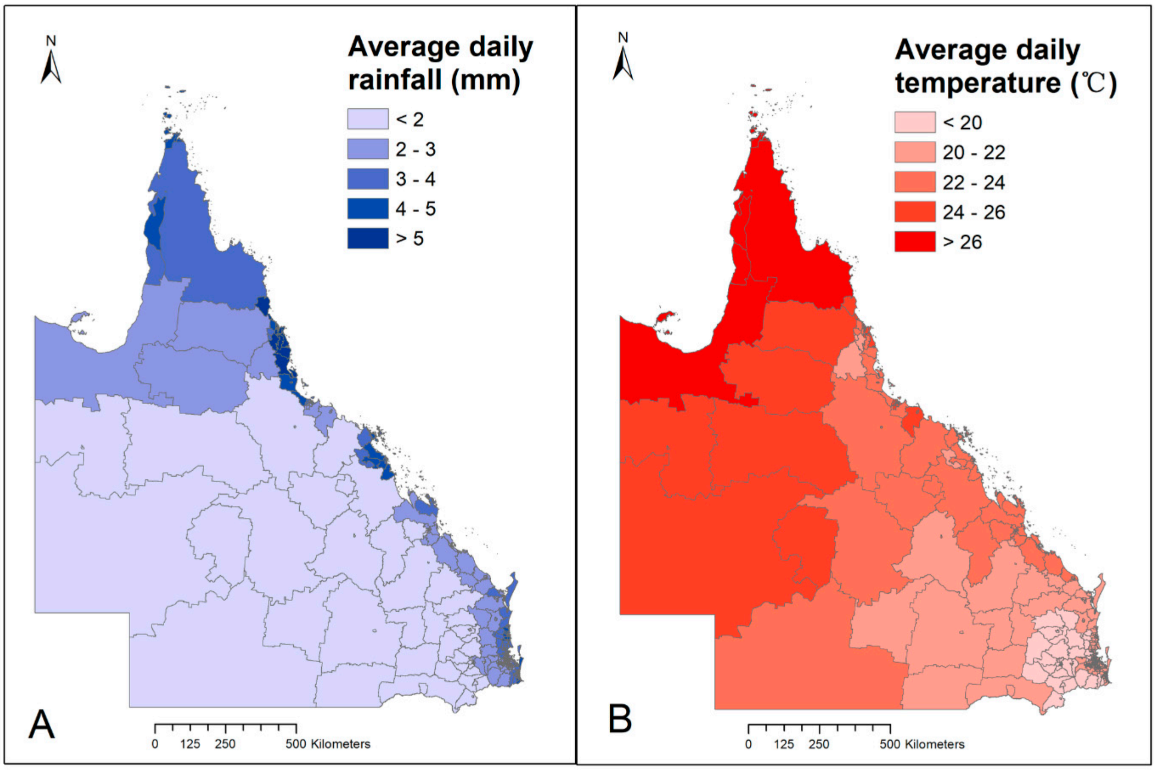

Figure S8. Average daily rainfall (mm, A) and daily temperature (°C, B) in SA2 areas of Queensland, 2001−2020.

Table S1. The top 100 most cited articles in the field of Rabies virus WOS around the world.

Rank	Article Title	Journal	Citations
1	Routes Of The Thalamus Through The History Of Neuroanatomy	Neuroscience And Biobehavioral Reviews	230
2	Liquid Biomolecular Condensates And Viral Lifecycles: Review And Perspectives	Viruses-Basel	223
3	Reactive Oxygen Species As Potential Antiviral Targets	Reviews In Medical Virology	181
4	The Microbiome Of Bat Guano: For What Is This Knowledge Important?	Applied Microbiology And Biotechnology	122
5	The Role Of A Mechanistic Host In Maintaining Arctic Rabies Variant Distributions: Assessment Of Functional Genetic Diversity In Alaskan Red Fox (Vulpes Vulpes)	Plos One	121
6	Characterizing And Evaluating The Zoonotic Potential Of Novel Viruses Discovered In Vampire Bats	Viruses-Basel	116
7	Cerebellar Projections To The Macaque Midbrain Tegmentum: Possible Near Response Connections	Visual Neuroscience	109
8	The Synaptic Inputs And Thalamic Projections Of Two Classes Of Layer 6 Corticothalamic Neurons In Primary Somatosensory Cortex Of The Mouse	Journal Of Comparative Neurology	109
9	The European Union One Health 2019 Zoonoses Report	Efsa Journal	107
10	Application Progress Of Rvg Peptides To Facilitate The Delivery Of Therapeutic Agents Into The Central Nervous System	Rsc Advances	106
11	Increased Callosal Connectivity In Reeler Mice Revealed By Brain-Wide Input Mapping Of Vip Neurons In Barrel Cortex	Cerebral Cortex	104
12	Can Transsynaptic Viral Strategies Be Used To Reveal Functional Aspects Of Neural Circuitry?	Journal Of Neuroscience Methods	101
13	Safe And Effective Two-In-One Replicon-And-Vlp Minispike Vaccine For Covid-19: Protection Of Mice After A Single Immunization	Plos Pathogens	97
14	Structural Insights Into Rna Polymerases Of Negative-Sense Rna Viruses	Nature Reviews Microbiology	97
15	Addressing Antiretroviral Drug Resistance With Host-Targeting Drugs-First Steps Towards Developing A Host-Targeting Hiv-1 Assembly Inhibitor	Viruses-Basel	90
16	Design And Validation Of Liposomal Apoe2 Gene Delivery System To Evade Blood-Brain Barrier For Effective Treatment Of Alzheimer's Disease	Molecular Pharmaceutics	86
17	Bioaccumulation Of Pathogen Exposure In Top Predators	Trends In Ecology & Evolution	85
18	Brain-Wide Mapping Of Presynaptic Inputs To Basolateral Amygdala Neurons	Journal Of Comparative Neurology	83
19	Lagos Bat Virus, An Under-Reported Rabies-Related Lyssavirus	Viruses-Basel	83
20	Viral Zoonoses Of National Importance In Ghana: Advancements And Opportunities For Enhancing Capacities For Early Detection And Response	Journal Of Tropical Medicine	83
21	Rabies: Presentation, Case Management And Therapy	Journal Of The Neurological Sciences	78
22	Tackling The Threat Of Rabies Reintroduction In Europe	Frontiers In Veterinary Science	75
23	Whole-Brain Mapping Of Direct Inputs To Dopamine D1 And D2 Receptor-Expressing Medium Spiny Neurons In The Posterior Dorsomedial Striatum	Eneuro	74
24	Whole-Brain Mapping The Direct Inputs Of Dorsal And Ventral Ca1 Projection Neurons	Frontiers In Neural Circuits	70
25	Comparison Of Lncrna And Mrna Expression In Mouse Brains Infected By A Wild-Type And A Lab-Attenuated Rabies Lyssavirus	Journal Of General Virology	70
26	Street Rabv Induces The Cholinergic Anti-Inflammatory Pathway In Human Monocyte-Derived Macrophages By Binding To Nachr Alpha 7	Frontiers In Immunology	69
27	Serological Responses Of Raccoons And Striped Skunks To Ontario Rabies Vaccine Bait In West Virginia During 2012-2016	Viruses-Basel	69

28	Relationships Between Fox Populations And Rabies Virus Spread In Northern Canada	Plos One	66
29	Hierarchy In Sensory Processing Reflected By Innervation Balance On Cortical Interneurons	Science Advances	65
30	Modified Live Distemper Vaccines Carry Low Mortality Risk For Captive African Wild Dogs, Lycaon Pictus	Journal Of Zoo And Wildlife Medicine	65
31	Glycosylation Is Required For The Neutralizing Activity Of Human Igg1 Antibodies Against Human Rabies Induced By Pre-Exposure Prophylaxis	Immunobiology	63
32	Aptamer And Rvg Functionalized Gold Nanorods For Targeted Photothermal Therapy Of Neurotropic Virus Infection In The Mouse Brain	Chemical Engineering Journal	62
33	Towards Development Of An Anti-Vampire Bat Vaccine For Rabies Management: Inoculation Of Vampire Bat Saliva Induces Immune-Mediated Resistance	Viruses-Basel	62
34	Multi-Annual Performance Evaluation Of Laboratories In Post-Mortem Diagnosis Of Animal Rabies: Which Techniques Lead To The Most Reliable Results In Practice?	Plos Neglected Tropical Diseases	61
35	Short Term Safety, Immunogenicity, And Reproductive Effects Of Combined Vaccination With Anti-Gnrh (Gonacon) And Rabies Vaccines In Female Feral Cats	Frontiers In Veterinary Science	60
36	Innate Inhibiting Proteins Enhance Expression And Immunogenicity Of Self-Amplifying Rna	Molecular Therapy	60
37	Rabies Virus Matrix Protein Targets Host Actin Cytoskeleton: A Protein-Protein Interaction Analysis	Pathogens And Disease	58
38	Chimeric Vlps Based On Hiv-1 Gag And A Fusion Rabies Glycoprotein Induce Specific Antibodies Against Rabies And Foot-And-Mouth Disease Virus	Vaccines	57
39	Monosynaptic Retrograde Tracing From Prelimbic Neuron Subpopulations Projecting To Either Nucleus Accumbens Core Or Rostromedial Tegmental Nucleus	Frontiers In Neural Circuits	57
40	Mrna-Based Sars-Cov-2 Vaccine Candidate Cvncov Induces High Levels Of Virus-Neutralising Antibodies And Mediates Protection In Rodents	Npj Vaccines	55
41	Change In The Single Amino Acid Site 83 In Rabies Virus Glycoprotein Enhances The Bbb Permeability And Reduces Viral Pathogenicity	Frontiers In Cell And Developmental Biology	55
42	Cost-Effectiveness Of The National Dog Rabies Prevention And Control Program In Mexico, 1990-2015	Plos Neglected Tropical Diseases	53
43	Relatedness And Genetic Structure Of Big Brownd Bat (Eptesicus Fuscus) Maternity Colonies In An Urban-Wildland Interface With Periodic Rabies Rabies Virus Outbreaks	Journal Of Wildlife Diseases	52
44	Artesunate And Dihydroartemisinin Inhibit Rabies Virus Replication	Virologica Sinica	52
45	The Successful Elimination Of Sylvatic Rabies Using Oral Vaccination Of Foxes In Slovenia	Viruses-Basel	52
46	Early Diagnosis Of Rabies Virus Infection By Rpa-Crispr Techniques In A Rat Model	Archives Of Virology	52
47	Evolutionary Analysis Of Rabies Virus Using The Partial Nucleoprotein And Glycoprotein Gene In Mumbai Region Of India	Journal Of General Virology	52
48	Rabies Virus Infection Is Associated With Alterations In The Expression Of Parvalbumin And Secretagogin In Mice Brain	Metabolic Brain Disease	51
49	Isolation And Characterization Of Cross-Reactive Human Monoclonal Antibodies That Potently Neutralize Australian Bat Lyssavirus Variants And Other Phylogroup 1 Lyssaviruses	Viruses-Basel	51
50	A Recombinase Polymerase Amplification Assay For Rapid Detection Of Rabies Virus	Scientific Reports	51

51	A Novel Oral Rabies Vaccine Enhances The Immunogenicity Through Increasing Dendritic Cells Activation And Germinal Center Formation By Expressing U-Omp19 In A Mouse Model	Emerging Microbes & Infections	51
52	Probability Of A Zoonotic Spillover With Seasonal Variation	Infectious Disease Modelling	51
53	Macrophage Membrane-Coated Nanocarriers Co-Modified By Rvg29 And Tpp Improve Brain Neuronal Mitochondria-Targeting And Therapeutic Efficacy In Alzheimer's Disease Mice	Bioactive Materials	50
54	Lentiviral Expression Of Rabies Virus Glycoprotein In The Rat Hippocampus Strengthens Synaptic Plasticity	Cellular And Molecular Neurobiology	50
55	Inactivated Rabies Virus Vectored Sars-Cov-2 Vaccine Prevents Disease In A Syrian Hamster Model	Plos Pathogens	49
56	Novel And Re-Emerging Zoonotic Viral Diseases In India During Last Two Decades: An Overview	Journal Of Pharmaceutical Research International	48
57	Delineating The Organization Of Projection Neuron Subsets In Primary Visual Cortex With Multiple Fluorescent Rabies Virus Tracing	Brain Structure & Function	47
58	Population Structure Of Whitefly (Bemisia Tabaci) And The Link Between Vector Dynamics And Seasonal Incidence Of Yellow Mosaic Disease In Blackgram (Vigna Mungo)	Entomologia Experimentalis Et Applicata	46
59	Neuronal Replacement In Stem Cell Therapy For Stroke: Filling The Gap	Frontiers In Cell And Developmental Biology	44
60	Investigation Of Protective Level Of Rabies Antibodies In Vaccinated Dogs In Chennai, India	Veterinary Record Open	42
61	Neglected Challenges In The Control Of Animal Rabies In China	One Health	42
62	Cerebral Cysticercosis In A Wild Bengal Tiger (Panthera Tigris Tigris) In Bhutan: A First Report In Non-Domestic Felids	International Journal For Parasitology-Parasites And Wildlife	42
63	Safety And Efficacy Of Rabies Immunoglobulin In Pediatric Patients With Suspected Exposure	Human Vaccines & Immunotherapeutics	41
64	Shared Odds Of Borrelia And Rabies Virus Exposure In Serbia	Pathogens	40
65	Rabies Virus Glycoprotein Enhances Spatial Memory Via The Pdz Binding Motif	Journal Of Neurovirology	40
66	Analyses Of Cell Death Mechanisms Related To Amino Acid Substitution At Position 95 In The Rabies Virus Matrix Protein	Journal Of General Virology	40
67	Serosurveillance Of Rabies Antibodies In Dogs In Mumbai Region By Using Indirect Elisa	Comparative Immunology Microbiology And Infectious Diseases	38
68	Full-Genome Sequences And Phylogenetic Analysis Of Archived Danish European Bat Lyssavirus 1 (Eblv-1) Emphasize A Higher Genetic Resolution And Spatial Segregation For Sublineage 1a	Viruses-Basel	38
69	Analysis Of Time Delayed Rabies Model In Human And Dog Populations With Controls	Afrika Matematika	38
70	Immunocontraceptive Potential Of A Gnrh Receptor-Based Fusion Recombinant Protein	Journal Of Genetic Engineering And Biotechnology	36
71	Begomoviruses Affecting Pulse And Vegetable Crops Are Unevenly Distributed In Distinct Agroecological Zones Of The Eastern India	Journal Of Phytopathology	36
72	Blood Metabolic And Hematology Parameters And Survivorship In Mice After Application Of The Rabies Challenge Virus Standard In Vaccine Potency Test	Acta Scientiae Veterinariae	34
73	A Preliminary Investigation Of Exposure To Rabies Virus In Selected Wildlife In The Kruger National Park, South Africa	Koedoe	34
74	Clofazimine: A Promising Inhibitor Of Rabies Virus	Frontiers In Pharmacology	33
75	Stool Serology: Development Of A Non-Invasive Immunological Method For The Detection Of Enterovirus-Specific Antibodies In Congo Gorilla Faeces	Microorganisms	32

76	Quantitative Characterization Of The T Cell Receptor Repertoires Of Human Immunized By Rabies Virus Vaccine	Human Vaccines & Immunotherapeutics	31
77	Rabies In Tunisia: A Spatio-Temporal Analysis In The Region Of Capbon-Nabeul	Acta Tropica	31
78	Knowledge, Attitudes And Practices Regarding Rabies Among Community Members: A Cross-Sectional Study In Songan Village, Bali, Indonesia	International Maritime Health	30
79	Ecology Of Arctic Rabies: 60 Years Of Disease Surveillance In The Warming Climate Of Northern Canada	Zoonoses And Public Health	29
80	Rabies And The Heart	Cardiology Research	28
81	Development Of Monoclonal Antibodies For Detection Of Conserved And Variable Epitopes Of Large Protein Of Rabies Virus	Viruses-Basel	28
82	Safety And Immunogenicity Of Rabies Vaccine As 4-Dose Essen Intramuscular Regimen For Post Exposure Prophylaxis: A Non-Randomized, Comparative Controlled Study	Human Vaccines & Immunotherapeutics	27
83	Genetic Identification Of A Rabies Virus From An Insectivorous Bat In An Urban Area Of Rio Grande Do Sul, Brazil	Brazilian Journal Of Microbiology	25
84	Proof-Of-Concept Of A Low-Dose Unmodified Mrna-Based Rabies Vaccine Formulated With Lipid Nanoparticles In Human Volunteers: A Phase 1 Trial	Vaccine	24
85	Long-Term Immunity And The Effect Of One Or Two Booster Doses With A Lyophilized Human Rabies Vaccine (Human Diploid Cells) At 10 Years Post Primary Vaccination In China	Human Vaccines & Immunotherapeutics	23
86	Evaluation Of The Effect Of Hydroethanolic Root Extract And Solvent Fractions Of Cyphostemma Adenocaule (Steud. Ex A. Rich) Descoings Ex Wild & Drummond On Cell-Mediated Immune Response And Blood Cell Count In Mice	Evidence-Based Complementary And Alternative Medicine	23
87	Systematic Booster After Rabies Pre-Exposure Prophylaxis To Alleviate Rabies Antibody Monitoring In Individuals At Risk Of Occupational Exposure	Vaccines	22
88	Rabies-Infected Dogs At Slaughterhouses: A Potential Risk Of Rabies Transmission Via Dog Trading And Butchering Activities In Vietnam	Zoonoses And Public Health	19
89	Lesions Associated With Bartonella Taylorii-Like Bacterium Infection In A Free-Ranging, Young-Of-The-Year Raccoon From Prince Edward Island, Canada	Journal Of Veterinary Diagnostic Investigation	19
90	Local Surveillance And Control Of Raccoon Rabies Virus In Striped Skunks (Mephitis Mephitis) In Southwestern New Brunswick, Canada	Journal Of Wildlife Diseases	18
91	Editorial: Mrna Vaccines And Future Epidemic, Pandemic, And Endemic Zoonotic Virus Infections	Medical Science Monitor	16
92	Rabies Virus Exposure In Wild Lowland Tapirs (Tapirus Terrestris) From Three Brazilian Biomes	Journal Of Wildlife Diseases	14
93	Safety, Pharmacokinetics, And Neutralizing Activity Of Syn023, A Mixture Of Two Novel Antirabies Monoclonal Antibodies Intended For Use In Postrabies Exposure Prophylaxis	Clinical Pharmacology In Drug Development	14
94	A Case Of Human Rabies With A Long Incubation Period In Wuhan	Idcases	14
95	Negligible Risk Of Rabies Importation In Dogs Thirty Days After Demonstration Of Adequate Serum Antibody Titer	Vaccine	13
96	Covid-19 Impacts On Agriculture And Food Systems Of India	International Journal Of Modern Agriculture	11
97	Development Of An Assay For Detecting The Residual Viable Virus In Inactivated Rabies Vaccine By Enzyme-Linked Immunosorbent Assay	Biologicals	9
98	The Serotonin Reuptake Inhibitor Fluoxetine Inhibits Sars-Cov-2 In Human Lung Tissue	Scientific Reports	9
99	Neurological Recovery With Serological Response In A Rabies Survivor On Long-Term Follow-Up	Tropical Doctor	5
100	Us-Japan Cooperative Medical Sciences Program: 22nd International Conference On Emerging Infectious Diseases In The Pacific Rim	Virology	0
